# Antiradical and Xanthine Oxidase Inhibitory Activity Evaluations of *Averrhoa bilimbi* L. Leaves and Tentative Identification of Bioactive Constituents through LC-QTOF-MS/MS and Molecular Docking Approach

**DOI:** 10.3390/antiox7100137

**Published:** 2018-10-08

**Authors:** Qamar Uddin Ahmed, Alhassan Muhammad Alhassan, Alfi Khatib, Syed Adnan Ali Shah, Muhammad Mahmudul Hasan, Murni Nazira Sarian

**Affiliations:** 1 Department of Pharmaceutical Chemistry, Kulliyyah of Pharmacy, International Islamic University Malaysia, Pahang DM, Kuantan 25200, Malaysia; alfikhatib@iium.edu.my (A.K.); mhasan222@gmail.com (M.M.H.); murninazira88@gmail.com (M.N.S.); 2 Department of Pharmaceutical and Medicinal Chemistry, Faculty of Pharmaceutical Sciences, Usmanu Danfodiyo University, Sokoto P.M.B. 2346, Nigeria; 3 Atta-ur-Rahman Institute for Natural Products Discovery (AuRIns), Universiti Teknologi MARA, Bandar Puncak Alam, Shah Alam 40450, Malaysia; syedadnan@salam.uitm.edu.my; 4 Faculty of Pharmacy, Puncak Alam Campus, Universiti Teknologi MARA, Bandar Puncak Alam 42300, Malaysia

**Keywords:** *Averrhoa bilimbi*, Oxalidaceae, DPPH, xanthine oxidase, LC-QTOF-MS/MS, molecular docking, tentative bioactive constituents

## Abstract

The objective of the present study was to investigate the antiradical and xanthine oxidase inhibitory effects of *Averrhoa bilimbi* leaves. Hence, crude methanolic leaves extract and its resultant fractions, namely hexane, chloroform, and n-butanol were evaluated for 2,2-diphenyl-1-picrylhydrazyl (DPPH) radical scavenging effect and xanthine oxidase inhibitory activity. The active constituents were tentatively identified through LC-QTOF-MS/MS and molecular docking approaches. The n-butanol fraction of *A. bilimbi* crude methanolic leaves extract displayed significant DPPH radical scavenging effect with IC_50_ (4.14 ± 0.21 μg/mL) (*p* < 0.05), as well as xanthine oxidase inhibitory activity with IC_50_ (64.84 ± 3.93 μg/mL) (*p* < 0.05). Afzelechin 3-*O*-alpha-l-rhamnopyranoside and cucumerin A were tentatively identified as possible metabolites that contribute to the antioxidant activity of the n-butanol fraction.

## 1. Introduction

Metabolic activity in the human body produces several reactive chemical species which are known as free radicals. Though some of these free radicals like superoxide (O^2−^), hydroxyl radical (OH), nitric oxide radical (NO), and hydrogen peroxide (H_2_O_2_) play beneficial roles in the maintenance of homeostasis, their presence in excessive amounts is often considered detrimental to the body system [1,2]. High levels of free radicals in the body are associated with increased risk of developing metabolic disorders like diabetes mellitus, and cardiovascular diseases [3,4,5]. Free radicals are frequently involved in the activation of signal transduction systems such as mitogen-activated protein kinases-dependent pathways which interfere with different gene expressions, homeostasis, and the development of diseases [5]. Similarly, increased activity of xanthine oxidase has been linked to the development of oxidative stress and metabolic diseases. There is significant correlation between increased xanthine oxidase activity and some metabolic syndrome markers viz. superoxide dismutase, abdominal circumference, high-sensitivity C-reactive protein content, and body mass indices [6]. Increased xanthine oxidase activity has also been implicated in the development of obesity which is considered a key factor in the pathogenesis of diabetes and cardiovascular diseases [7].

Plants have the inherent ability to biosynthesize several compounds with antioxidant properties which could be explored in the search for a better antioxidant agent for the enhancement of health and prevention of diseases [8,9]. *Averrhoa bilimbi* (family Oxalidaceae) is an Asian medicinal plant commonly known in the Malay language as “belimbing buluh”. *A. bilimbi* has been reported to possess significant antioxidant and antidiabetic properties [10]. Topical administration of *A. bilimbi* crude leaves extract has been reported to impede ultraviolet light-induced oxidative damage in albino mice [11]. Oral administration of the methanolic extract (250 and 500 mg/kg,) has been reported to prevent CCl_4_-induced hepatic damage in experimental animal model [12]. Crude ethanol and water extracts, as well as the semi-purified butanol fraction of *A. bilimbi* leaves have been reported to display significant (*p* < 0.05) antihyperglycemic activity, i.e., comparable to metformin in streptozotocin-induced diabetic rats [13,14,15]. However, there is a paucity of information on the phytoconstituents of this plant that could be responsible for its antioxidant properties. Free radicals and xanthine oxidase are believed to be associated with the development of oxidative stress and related metabolic disorders [3,4,5,6,7]. Hence, this study investigates the in vitro antiradical and xanthine oxidase inhibitory effects of *A. bilimbi* leaves. An attempt has also been made to identify the active constituents of *A. bilimbi* leaves through LC-QTOF-MS/MS analysis and the molecular docking approach.

## 2. Materials and Methods

### 2.1. Materials

Allopurinol, xanthine, xanthine oxidase, potassium di-hydrogen phosphate (KH_2_PO_4_), di-potassium hydrogen phosphate (K_2_HPO_4_), DPPH, and ascorbic acid were purchased from Sigma-Aldrich Chemicals (St. Louis, MO, USA). Dimethylsulphoxide (DMSO), hydrochloric acid (HCl), absolute ethanol, methanol, chloroform, n-butanol, hexane, and other reagents of analytical grade were obtained from Merck (Darmstadt, Germany).

### 2.2. Plant Collection and Processing

Fresh leaves (1 kg) of *A. bilimbi* were obtained in July 2016 from the garden at Indera Makhota in the neighbourhood of International Islamic University Malaysia (IIUM), Kuantan Campus. The plant was identified and authenticated by the taxonomist at Taman Pertanian, Indera Makhota, 25200 Kuantan, Pahang Darul Makmur, Malaysia. Subsequently, the sample was deposited in the herbarium of Kulliyyah of Pharmacy, IIUM, Kuantan, Pahang DM, Malaysia (voucher no: NMPC-QAAB-12). The fresh plant material was initially dried in a PROTECH laboratory air dryer (FDD-720-Malaysia) at 45 °C. Thereafter, the dried plant material was pulverised into a powdered form using Fritsch Universal Cutting Mill-Pulverisette 19-Germany. The powdered material was kept in airtight polythene bags at 4 °C prior to further use.

### 2.3. Extraction and Fractionation

Total leaf extract was prepared by soaking powdered leaves (350 g) in methanol (2 L) at room temperature for 48 h, followed by filtration and concentration of the filtrate under reduced pressure using a rotary evaporator (Buchi, Switzerland). The entire procedure was repeated 3 times to yield 40.5 g (11.6%) of crude methanolic leaves extract. Afterwards, the dried crude methanolic leaves extract was subjected to liquid-liquid fractionation. The extract was first suspended in 10% aqueous ethanol, then transferred into the separating funnel and sequentially extracted with hexane, chloroform, n-butanol, and double distilled water to afford hexane fraction (11.67 g), chloroform fraction (8.26 g), n-butanol (8.30 g), and aqueous fraction (12.14 g), respectively.

### 2.4. Preliminary Phytochemical Screening

The crude methanolic leaves extract of *A. bilimbi* was initially subjected to various phytochemical tests to detect the occurrence of a different class of secondary metabolites which are associated with the existence of flavonoids (Shinoda test) [16], terpenoids (Salkowski test), saponins (frothing test) [17], alkaloids (Dragendorff’s and Mayer’s tests) and anthraquinones (free and combined) [18].

### 2.5. LC-QTOF-MS and LC-QTOF-MS/MS Analyses of Bioactive Constituents of n-Butanol Fraction

The LC-QTOF-MS and LC-QTOF-MS/MS analyses of the active n-butanol fraction, which showed significant antiradical (DPPH radical scavenging activity) and xanthine oxidase inhibitory effects, were carried out using an Agilent 1290 Infinity Ultra high performance liquid chromatographic system (LC) (Agilent, Santa Clara, CA, USA) coupled to an Agilent 6520 Accurate-Mass Q-TOF mass spectrometer (Agilent, Santa Clara, CA, USA) equipped with dual electrospray ionization (ESI) source. The n-butanol fraction was prepared at a concentration of 0.01 mg/mL in methanol, and was first filtered using a 0.45 µm nylon syringe filter before an aliquot was collected (3.0 µL) and injected into the LC system. The sample was separated on a reverse phase column [Agilent Zorbax Eclipse XDB-C18, Narrow-Bore 2.1 × 150 mm, 3.5 micron (P/N: 930990-902)] (Agilent, Santa Clara, CA, USA). The mobile phase included A–0.1% formic acid in water, and B–0.1% formic acid in acetonitrile. The column was eluted using a gradient mode under the following conditions. Auto sampler temperature: 6 °C; column temperature: 25 °C; flow rate: 0.5 mL/min; linear gradient from 5% B to 100% B from 0 min to 25 min. Full scan mass spectrometry was carried out using ESI positive ionization mode resulting in full mass spectrum with mass range (*m*/*z*) of 100–3200. The system workstation was equipped with a spectra library of several compounds which was used in the tentative identification of major, possibly bioactive constituents based on their accurate mass and elemental composition.

### 2.6. DPPH Free Radical Scavenging Activity

The DPPH (2,2-diphenyl-1-picrylhydrazyl) free radical scavenging activity of *A. bilimbi* crude methanolic leaves extract, and its fractions viz. hexane, chloroform, and n-butanol fractions, was evaluated according to the protocol described by Nickavar et al. and Barontini et al. [19,20] with some modifications. Briefly, 1 mL of different concentrations of test samples in methanol (4.8 μg/mL–1000 μg/mL) were prepared and respectively treated with 2 mL of 0.1 mM of freshly prepared DPPH solution in methanol, and finally, diluted using 1 mL of deionized water. The mixture was allowed to stay in an incubator at 30 °C for 30 min and absorbance at 517 nm was recorded using a microplate reader (Infinite M200 Nanoquant Tecan, Männedorf, Switzerland). Methanol was used as the blank, whereas DPPH, methanol, and water (2:1:1) served as controls. Ascorbic acid was used as positive standard and IC_50_ values in μg/mL were determined. The IC_50_ values were calculated by the linear regression of plots. The percentage DPPH free stable radical scavenging activity was computed using the following equation:

% radical scavenging activity = (*Abs*_control_ − *Abs*_sample_/*Abs*_control_) × 100.

where, *Abs*_control_ = absorbance of control, *Abs*_sample_ = absorbance of extract, fraction or ascorbic acid.

### 2.7. Xanthine Oxidase Inhibitory Activity Assay

The xanthine oxidase inhibitory activity of *A. bilimbi* crude methanolic leaves extract and its fractions viz. hexane, chloroform and n-butanol fractions was determined by following the protocol described by Thiombiano et al. [21]. The assay was carried out in 96 well plates. The reaction mixture was prepared in such a way that it contains 50 μL of test solution together with 30 μL of freshly xanthine oxidase solution (0.2 units/mL). The assay mixture was pre-incubated at 37 °C for 15 min. Thereafter, the reaction was initiated by the addition of 60 μL of substrate solution (0.15 mM of xanthine). The mixture was placed in the incubator set at 37 °C and allowed to stay for 30 min. The reaction was terminated by the addition of 50 μL of 0.5 M HCl. A known potent xanthine oxidase inhibitor, viz. allopurinol, was used as positive control. The absorbance at 295 nm was measured using microplate reader (Infinite M200 Nanoquant Tecan, Tecan Group Ltd., Männedorf, Switzerland). The percentage of xanthine oxidase inhibition was calculated using the following equation:

% xanthine oxidase inhibition = (*Abs*_control_ − *Abs*_sample_/*Abs*_control_) × 100.

where, *Abs*_control_ = activity of enzyme without extract/fraction, and *Abs*_sample_ = enzyme activity in presence of extract/fraction or allopurinol.

### 2.8. Molecular Docking

The phenolic compounds identified through analysis in the n-butanol fraction of *A. bilimbi* crude leaves methanolic extract were considered as possible molecules that contribute to the antioxidant properties. Hence, they were docked into the active site of the crystal structure of xanthine oxidase. The 3D structure for bovine xanthine oxidase, co-crystallized with standard inhibitor, quercetin (PDB ID: 3NVY), was obtained from a protein data bank (PDB) (http://www.rcsb.org/) [22]. Software including Molecular Graphics Library MGLTools 1.5.6 (The Scripps Research Institute, San Diego, CA, USA), AutoDock Tools 4.2 (www.scripps.edu), (The Scripps Research Institute, San Diego, CA, USA) [23], and Discovery Studio Visualizer 4.0 (www.accelrys.com) Bovia, San Diego, CA, USA) [24] were used for the molecular docking experiments. Crystallographic waters and co-crystallized ligand were removed, and polar hydrogens were added to a macromolecule using AutoDock 4.2, after which the structure was saved in the PDBQT file format that contains a protein structure with hydrogen in all polar residues. The 2D structures of ligands were drawn through ChemDraw^®^ (PerkinElmer, Waltham, MA, USA) and converted to 3D format using ChemBio3D Ultra^®^ 14 Suit (PerkinElmer, Waltham, MA, USA), which were further subjected to energy minimization using molecular mechanics 2 (MM2) force field and saved in PDB file format. The ligands were then prepared for docking by computing the charges, and the structures were saved in PDBQT file format via AutoDock 4.2. Molecular docking experiment of ligands was performed following the protocol described by Zhang et al. [25]. The size and center of the coordinates of the grid box was first validated by re-docking the co-crystallized ligand into the active sites of the receptor. The rotational bonds of the ligands were considered to be flexible, while those of the receptor were kept rigid. A grid box was centered on binding site of the co-crystallized ligand. The box size was set to 50, 50 and 50 Å^3^ (x, y and z, respectively) and the grid spacing to 0.375 Å. The grid maps for atoms were calculated and genetic algorithm (GA) was used for searching; the population size was set to 150, 100 runs, and 5 million energy evaluations. The ligands (identified compounds) were then docked into the active site of the enzyme using the grid box parameter obtained from the re-docking of co-crystallized ligand as reference.

### 2.9. Statistical Analysis

All measurements were carried out in triplicate. The 50% inhibitory concentration (IC_50_) was calculated by nonlinear regression analysis and expressed as mean ± standard deviation (SD). Means were compared for statistically significant differences by one-way analysis of variance (ANOVA) using GraphPad Prism 7 (La Jolla, San Diego, CA, USA) with a 95% confidence interval.

## 3. Results and Discussion

The results of the various phytochemical tests carried out on *A. bilimbi* crude methanolic leaves extract are shown in Table 1. The results indicate the presence of alkaloids, flavonoids, terpenoids, and saponins (which have positive inference), while the extract showed negative results for the presence of free and combined anthraquinones.

### 3.1. DPPH Radical Scavenging Activity

The DPPH radical exhibits absorption maxima at *λ* = 517 nm in its stable state. This absorption decreases when the stable radical undergoes reduction to a hydrazine derivative in the presence of antioxidant agent; consequently, the purple colour of the DPPH reagent is changed to pale yellow [26]. The reduction is achieved either through the donation of a hydrogen atom to DPPH, or the removal of oxygen. The results of the DPPH radical scavenging activity which demonstrated the antiradical effect for crude methanolic extract and its subsequent fractions, as well as reference standard (ascorbic acid), are shown in Table 2.

The results of the DPPH assay showed that the n-butanol fraction displays the highest activity, followed by the crude methanolic leaves extract and the chloroform fraction, while the hexane fraction did not show any significant radical scavenging activity. The radical scavenging activity displayed by the n-butanol fraction was comparable to that of the ascorbic acid, which was used as reference standard. The substantial antioxidant activity of the n-butanol fraction could be due to the presence of phenolic constituents which are frequently soluble in polar solvents and are known to display significant radical scavenging activity [8,27]. The IC_50_ value for the chloroform (13.44 ± 1.00) fraction also signifies a significant radical scavenging effect, which suggests the presence of some active constituents.

### 3.2. Xanthine Oxidase Inhibitory Activity

Xanthine oxidase as the name implies is an enzyme that is responsible for the conversion of xanthine and hypoxanthine to uric acid through oxidation reaction. Increased activity of xanthine oxidase leads to over production of uric acid and resultant hyperuricemia [28]. Increased levels of uric acid in the blood are implicated in the development of gouty arthritis, as well as increased reactive oxygen species, which are involved in various pathological processes [29]. Table 3 shows the result for the xanthine oxidase inhibitory activity of *A. bilimbi* crude methanolic leaves extract and its resultant fractions. The n-butanol fraction displayed significant (*p* < 0.05) activity with 50% effective concentration (IC_50_) of 64.84 ± 3.93 μg/mL, while the hexane and chloroform fractions displayed very weak inhibitory activities. The xanthine oxidase inhibitory activity observed with this fraction suggests that it contains substantial amount of phytoconstituents with xanthine oxidase inhibitory potential.

Xanthine oxidase is present in large quantities in the liver, where it plays vital role in purine nucleotide metabolism. The activity of xanthine oxidase has been linked to the generation of reactive oxygen species such as superoxide (O^2−^), which is known to play vital roles in the development and progression of important pathological conditions like diabetes mellitus and related complications [30]. This result suggests that inhibition of xanthine oxidase could be part of the mechanism of *A. bilimbi* activity against oxidative tissue damage.

### 3.3. LC-QTOF-MS Analysis of n-Butanol Fraction of Averrhoa bilimbi Crude Methanolic Leaves Extract

**Figure 1:**
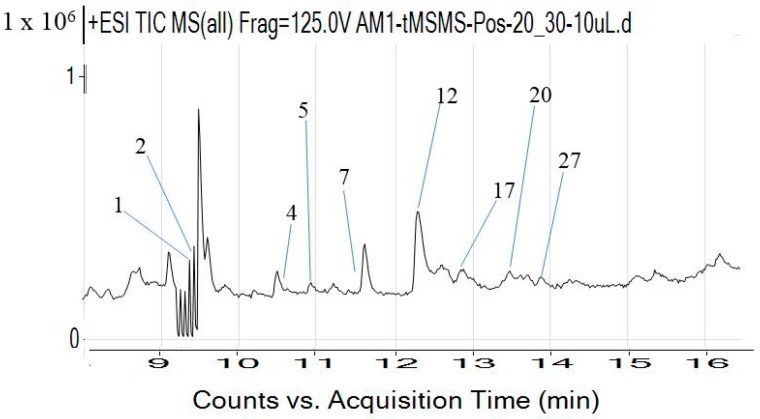
LC-MS spectrum of n-butanol fraction of *Averrhoa bilimbi* crude methanolic leaves extract (For compounds and retention time, please refer to Table 4).

**Figure 2:**
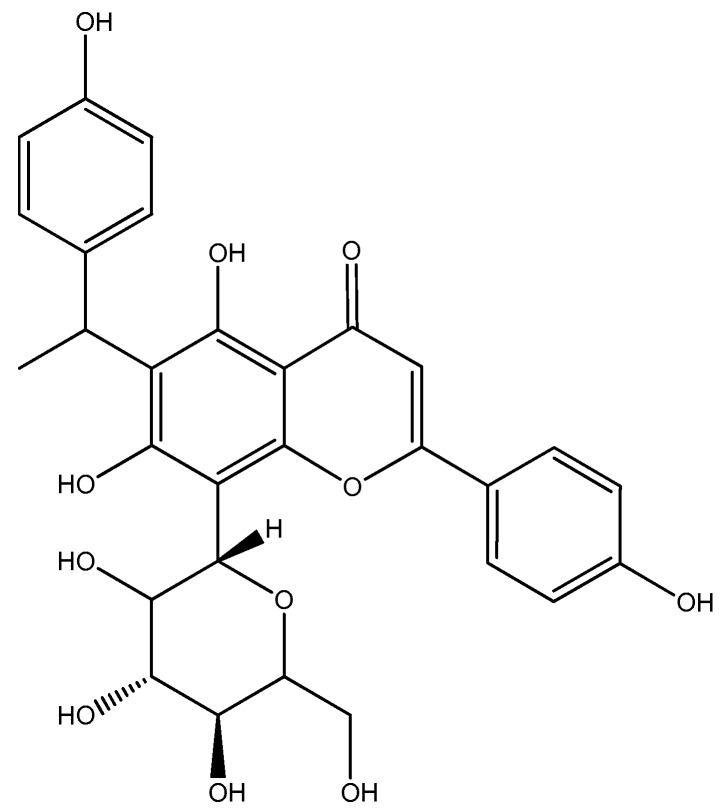
Tentative structure of 5,7,4′-trihydroxy-6-(1-ethyl-4-hydroxyphenyl)flavone-8-C-glucoside (cucumerin A).

**Figure 3:**
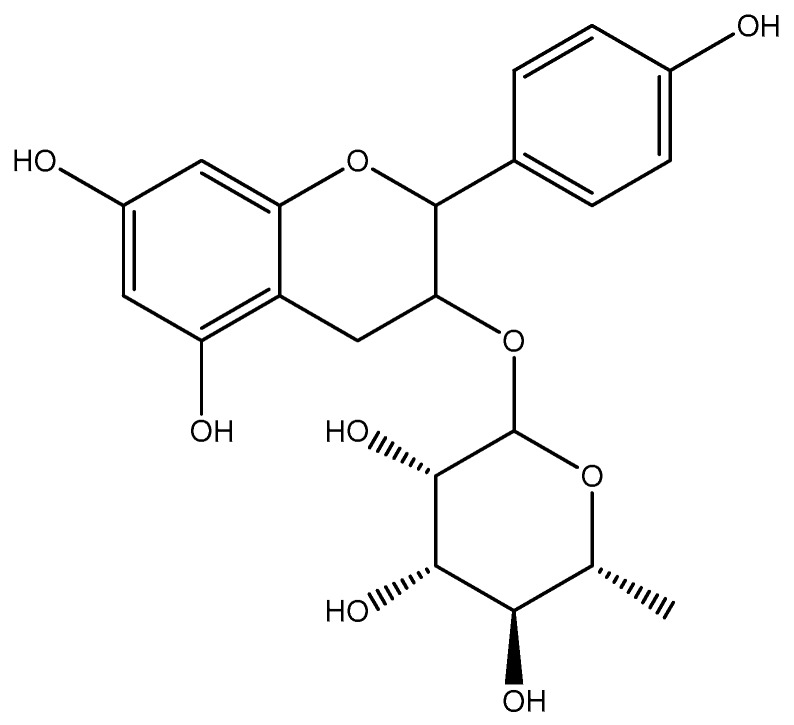
Tentative structure of afzelechin 3-*O*-alpha-l-rhamnopyranoside.

The results of the LC-QTOF-MS analysis of active n-butanol fraction of *A. bilimbi* crude methanolic leaves extract are shown in Figure 1 and Table 4. Several compounds which were detected in the active n-butanol fraction have been identified from the LC-MS compound library by matching their accurate mass. There were also some unknown compounds that could not be identified because they were not present in the compound database. The list of identified compounds include two phenolic constituents, namely, 5,7,4′-trihydroxy-6-(1-ethyl-4-hydroxyphenyl) flavone-8-C-glucoside (cucumerin A) (Figure 2) and afzelechin 3-*O*-alpha-l-rhamnopyranoside (Figure 3) which could partly be responsible for the antiradical and xanthine oxidase inhibitory activity of the n-butanol fraction of the *A. bilimbi* crude methanolic leaves extract.

The two compounds, viz. cucumerin A and afzelechin 3-*O*-alpha-l-rhamnopyranoside, are flavonoid glycosides. The antioxidant activities of flavonoid glycosides have been well studied. Based on our literature search, we discovered that there is no available literature focusing specifically on the radical scavenging effect or xanthine oxidase inhibitory activity of afzelechin 3-*O*-alpha-l-rhamnopyranoside and cucumerin A. However, previous research findings have shown that related flavonoid glycosides displayed significant antioxidant potential through DPPH radical scavenging effect and xanthine oxidase inhibitory activity [31,32]. Thus, it can be rightly construed that the antioxidant activity of the n-butanol fraction of *A. bilimbi* crude methanolic leaves extract could partly be due to the presence of phytoconstituents which were identified through the LC-QTOF-MS analysis.

### 3.4. Identification of Compounds Structures through Fragmentation Analysis (LC-QTOF-MS/MS) 

**Figure 4:**
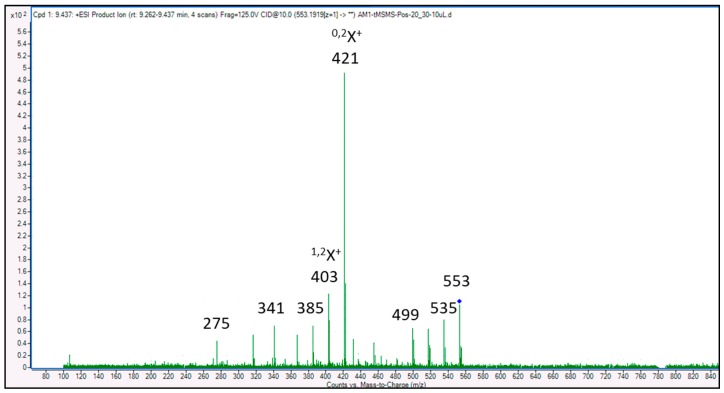
MS/MS spectrum of cucumerin A.

**Figure 5:**
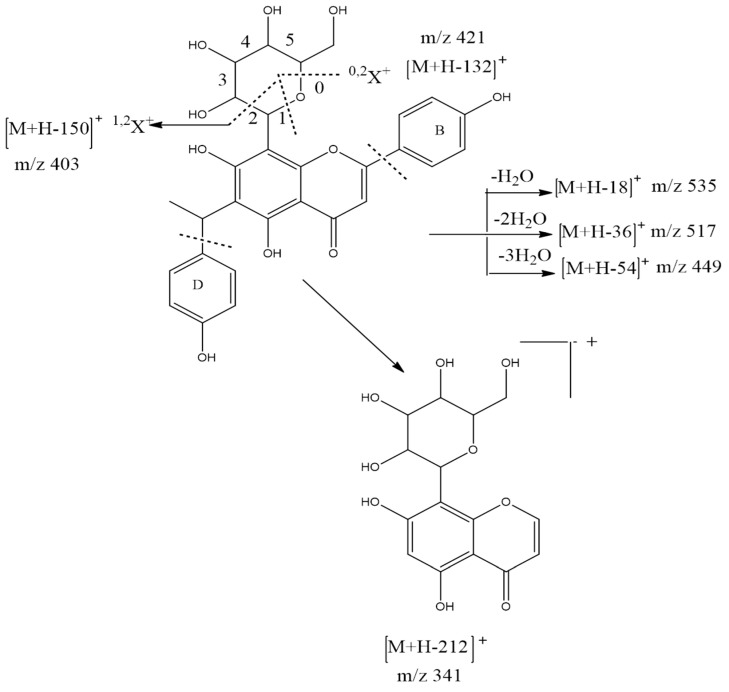
Fragmentation pattern of cucumerin A.

**Figure 6:**
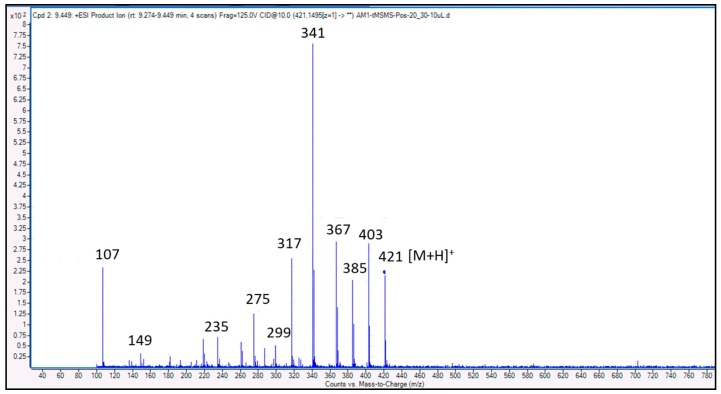
MS/MS spectrum of afzelechin 3-*O*-alpha-l-rhamnopyranoside.

**Figure 7:**
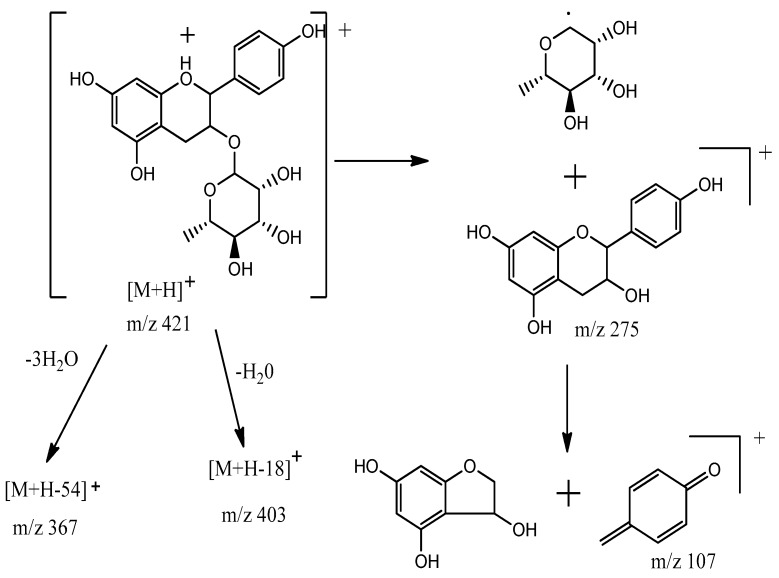
Fragmentation pattern of afzelechin 3-*O*-alpha-l-rhamnopyranoside.

A Q-TOF LC-MS system was used to analyze samples with some modifications [33]. LC-QTOF-MS/MS analysis was carried out to study the fragmentation pattern of 5,7,4′-trihydroxy-6-(1-ethyl-4-hydroxyphenyl)flavone-8-glucoside (cucumerin A) (Figure 4 and Figure 5) and afzelechin 3-*O*-alpha-l-rhamnopyranoside (Figure 6 and Figure 7), in order to further characterize the structure of these compounds. The MS/MS spectrum and fragmentation pattern of cucumerin A are shown in Figure 4 and Figure 5, respectively. The spectrum shows the presence of fragment ions that are the characteristic of C-glycosides. The ion peaks at *m*/*z* 403 and *m*/*z* of 421 representing [M + H-150]^+^ and [M + H-132]^+^ are typical of flavonoids with C-glycosides [34]. The peak at *m*/*z* 421 (base peak) represents the most stable ion fragment. In addition, a neutral loss of water molecules was observed, which produced fragment ions at [M + H-18]^+^, [M + H-36]^+^ and [M + H-54]^+^. A peak at *m*/*z* 341 [M + H-132]^+^, which indicates the loss of the B ring of the flavone skeleton and 1-ethyl-4-hydroxyphenyl ring attached to C-6 of the compound, further confirmed its structure. Based on this fragmentation pattern, we resolve the structure of this compound as 5,7,4′-trihydroxy-6-(1-ethyl-4-hydroxyphenyl) flavone-8-glucoside (cucumerin A). Figure 6 and Figure 7 show the MS/MS spectrum and fragmentation pattern of afzelechin 3-*O*-alpha-l-rhamnopyranoside, respectively. The peak at *m*/*z* 275 represents the aglycone afzelechin ion, which was produced from the loss of the glycone moiety (O-rhamnosyl). This form of fragmentation pattern is characteristic of flavone-O-glycosides [35]. The aglycone ion underwent further fragmentation to yield an ion peak at *m*/*z* 107, which is characteristic of the flavan. The spectrum also revealed the loss of water molecules with peaks at *m*/*z* 403 and 367 representing [M + H-18]^+^ and [M + H-54]^+^. Based on this information, we tentatively identified this compound as afzelechin 3-*O*-alpha-l-rhamnopyranoside.

### 3.5. Molecular Docking

**Figure 8:**
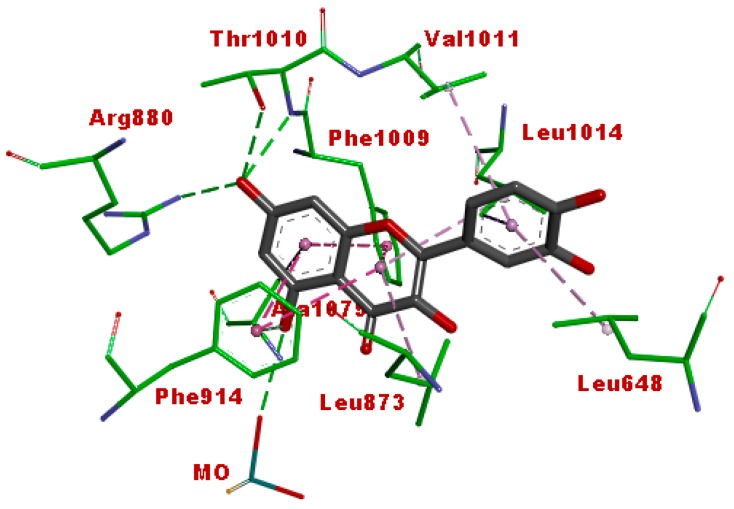
Binding interaction of quercetin (co-crystallized ligand) with the active site residues of xanthine oxidase (protein data bank (PDB) code: 3NVY). Green and purple dashes represent hydrogen-bonding and hydrophobic interactions, respectively.

The structure of vertebrate xanthine oxidase exists as a homodimer with each of its functional unit having four redox sites, viz. an active-site molybdenum center, a pair of spinach ferredoxin-like [2Fe-2S] clusters, and a redox cofactor as flavin adenine dinucleotide (FAD) [36]. Figure 8 shows the active site of bovine xanthine oxidase (PDB code: 3NVY) with quercetin as the co-crystallized ligand. The amino acid residues in the active site include Arg880, Thr1010, Glu808, Phe1009, Ala1079, Phe914, Val1011, Leu1014, Leu873, and Leu648. The 7-OH of quercetin formed hydrogen-bonding interactions with Arg880 and Thr1010. The benzopyran ring displayed pi-pi stacking interactions with Phe914 and Phe1009, while 5-OH formed hydrogen-bonding interaction with the Mo. These interactions are considered as important requirements for the inhibitory activity of flavonoids against xanthine oxidase [36].

**Figure 9:**
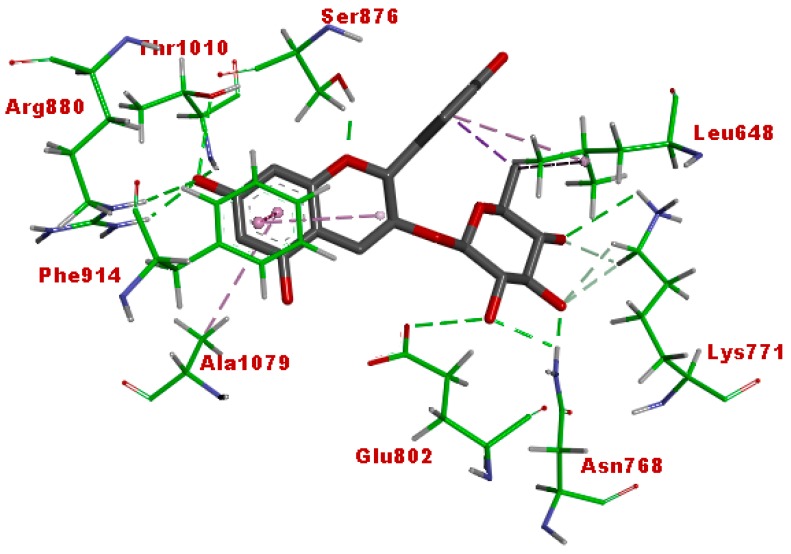
Binding interaction of afzelechin 3-*O*-alpha-l-rhamnoside with the active site residues of xanthine oxidase (PDB code: 3NVY). Green and purple dashes represent hydrogen-bonding and hydrophobic interactions, respectively.

Figure 9 shows the binding pose of the afzelechin 3-*O*-alpha-l-rhamnopyranoside in the active site of xanthine oxidase. This compound displayed a binding pose similar to quercetin. The 7-OH formed hydrogen-bonding interactions with Arg880 and Thr1010, while the benzopyran ring exhibited pi-pi stacking interaction with Phe914. These interactions suggest that this compound may exert inhibitory activity against the enzyme. A pi-hydrophobic interaction was observed between the aromatic ring B of the flavan and Leu648. The glycone moiety displayed extensive hydrogen-bonding interactions with Lys771, Glu802, and Asn768, which may also play a role in the xanthine oxidase inhibitory activity of this molecule.

**Figure 10:**
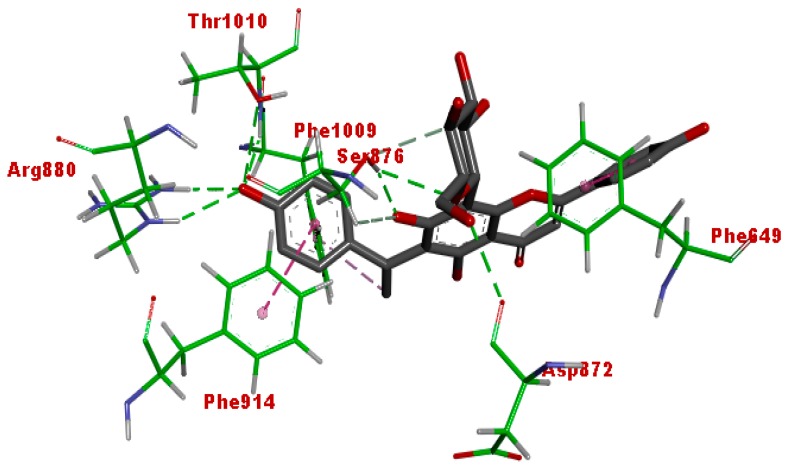
Binding interaction of cucumerin A with the active site of xanthine oxidase (PDB code: 3NVY). Green and purple dashes represent hydrogen-bonding and hydrophobic interactions, respectively.

**Figure 11:**
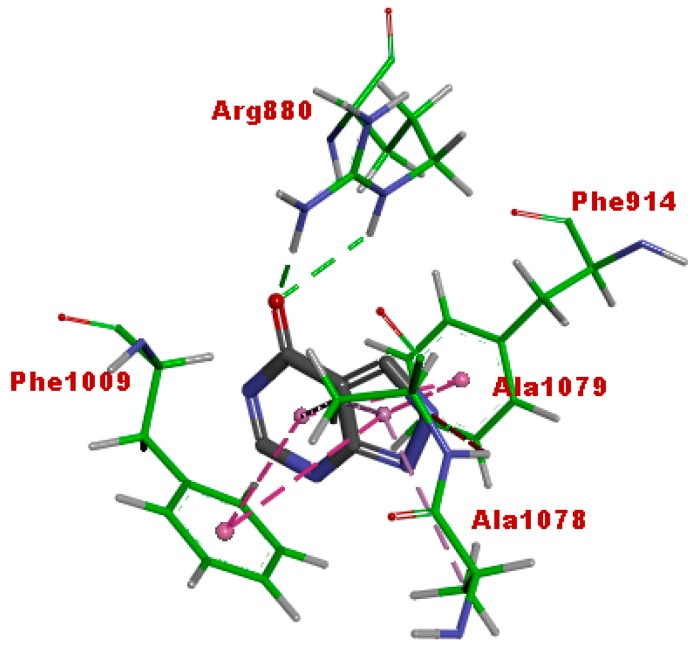
Binding interactions of allopurinol with the active site residues of xanthine oxidase (PDB code: 3NVY). Green and purple dashes represent hydrogen-bonding and hydrophobic interactions, respectively.

The binding pose of cucumerin A in the active site of xanthine oxidase is shown in Figure 10. Cucumerin A displayed similar interaction with quercetin and afzelechin. The 4-OH of the phenyl ethyl group binds into the catalytic pocket, exhibiting hydrogen bonding with Arg880 and Thr1010, as well as pi-pi stacking with Phe914 and Phe1010. The 7-OH of the flavonoid ring formed hydrogen bonding with Ser879, while the glycone moiety interacted with Asp872 as well as Ser876 through hydrogen bonding. These interactions show that cucumerin A could also contribute to the xanthine oxidase inhibitory effect of the butanol fraction of the *A. bilimbi* crude methanolic leaves extract. Figure 11 shows the binding pose of allopurinol, a known inhibitor of xanthine oxidase. The C=O group displayed hydrogen-bonding interaction with Arg 880, while the aromatic ring was stacked by pi-pi hydrophobic interactions with Phe914 and Phe1009. Additional hydrophobic interactions were observed with Ala 1078 and Ala1089. These interactions were similar to what was observed in the case of quercetin, cucumerin A, and afzelechin 3-*O*-alpha-l-rhamnopyranoside. However, it did not exhibit a hydrogen-bonding interaction with Thr1010, as observed with the aforementioned three flavonoids.

Table 5 shows the docking score of the two phenolic compounds, the co-crystallized ligand and allopurinol. The lower the free binding energy, the greater the interactions of the compound with the enzyme active site residues, which may consequently lead to better inhibitory effect. Cucumerin A and afzelechin 3-*O*-alpha-l-rhamnopyranoside have lower free binding energies than both quercetin and allopurinol. This further suggests that these two compounds could be responsible for the significant xanthine oxidase inhibitory effect of *A. bilimbi* leaves.

## 4. Conclusions

*A. bilimbi* is an important medicinal plant widely used in traditional medicine. The findings of this research study demonstrate that the leaves of *A. bilimbi* possess strong in-vitro antioxidant capacity, which is partly displayed through scavenging of free radicals and inhibition of xanthine oxidase activity. This antioxidant property was found to be significant in the n-butanol fraction, as revealed by the IC_50_ value. LC-MS-QTOF analysis, as well as molecular docking studies, led to the tentative identification of 5,7,4′-trihydroxy-6-(1-ethyl-4-hydroxyphenyl) flavone-8-glucoside (cucumerin A) and afzelechin 3-*O*-alpha-l-rhamnopyranoside as the compounds that are likely to be responsible for the antioxidant effect of this plant. However, other phytoconstituents which were not present in the library of the LC-MS system could not be identified from their accurate mass, and may also play some roles. Further research is needed to obtain such bioactive compounds in pure form for complete pharmacological evaluations.

## Figures and Tables

**Table 1:** Phytochemical screening for chemical class identification of *Averrhoa bilimbi* crude methanolic leaves extract.

Bioactive	Test/Procedure	Observation	Inference
Alkaloid	Dragendorff’s reagent	Orange red ppt.	+
	Mayer’s reagent	Cream ppt.	+
Saponin	Frothing test	Frothing	++
Terpenoid	Salkowski test:		
	Chloroform + H_2_SO_4_	Brownish red ring at the junction	+++
Flavonoid	Shinoda test:		
	Flavones: Mg filling	Reddish brown	++
	Flavonols: Zn pellet	Amber	++
Free anthraquinone	Chloroform + NH_3_ (10%)	No change from dirty green	−
Combined anthraquinone	10% HCl + Chloroform	No change from colourless	−

+++: Strong intensity reaction, ++: Medium intensity reaction, +: Weak intensity reaction, −: Undetected.

**Table 2:** Free radical scavenging (DPPH) activity of *Averrhoa bilimbi* crude methanolic leaves extract and its fractions.

Sample	IC_50_ (μg/mL)
Crude methanolic extract	10.53 ± 0.72 *
Hexane fraction	>1000
Chloroform fraction	13.44 ± 1.00 *
n-Butanol fraction	4.14 ± 0.21 *
Ascorbic acid (positive control)	5.52 ± 0.29

Values are mean of triplicate experiments ± standard deviation. The results were analysed using one way Analysis of variance (ANOVA). The superscript (*) indicates significant activity (*p* < 0.05). IC_50_ represents the concentration required to scavenge 50% of available radicals. The IC_50_ values were calculated by linear regression of plots.

**Table 3:** IC_50_ for xanthine oxidase inhibitory activity of *Averrhoa bilimbi* crude methanolic leaves extract and its fractions.

Sample	IC_50_ μg/mL
Crude methanolic leaves extract	>1000
Hexane fraction	>1000
Chloroform fraction	>1000
n-Butanol fraction	64.84 ± 3.93 *
Allopurinol (positive control)	16.21 ± 0.91

Values are mean of triplicate experimental results with standard deviation. The results were analysed using one way ANOVA. The symbol asterisk (*) indicates significant activity (*p* < 0.05).

**Table 4:** Results of LC-QTOF-MS analysis of n-butanol fraction.

Number	Tentative Compounds	Retention Time	Molecular Formula/Molecular Weight (M^+^)
1	5,7,4′-Trihydroxy-6-(1-ethyl-4-hydroxyphenyl)flavone-8-C-glucoside (Cucumerin A)	9.305	C_29_H_28_O_11_/552.185
2	Afzelechin 3-*O*-alpha-l-rhamnopyranoside	9.305	C_21_H_24_O_9_/420.142
3	Ethyl 3-(*N*-butylacetamido)propionate	9.997	C_11_H_21_NO_3_/215.152
4	Elaeokanine C	10.749	C_12_H_21_NO_2_/211.156
5	2-Ethyl-dodecanoic acid	10.958	C_14_H_2__8_O_2_/228.208
6	Isoavocadienofuran	11.063	C_17_H_2__6_O/246.198
7	(5alpha,8beta,9beta)-5,9-Epoxy-3,6-megastigmadien-8-ol	11.451	C_13_H_2__0_O_2_/208.144
8	Diglycidyl resorcinol ether	12.120	C_12_H_14_O_4_/222.090
9	19-Hydroxycinnzeylanol 19-glucoside	12.212	C_26_H_42_O_13_/562.264
10	Xestoaminol C	12.236	C_14_H_31_NO/229.240
11	Phytosphingosine	12.266	C_18_H_39_NO_3_/317.293
12	2-Hydroxyhexadecanoic acid	12.334	C_16_H_32_O_3_/272.236
13	Pentadecanal	12.408	C_15_H_30_O/226.230
14	Anapheline	12.707	C_13_H_24_N_2_O/224.188
15	Palmitic amide	12.713	C_16_H_33_NO/255.256
16	Tetradecylamine	12.769	C_14_H_31_N/213.246
17	Pentadecanoyl-EA	12.825	C_17_H_35_NO_2_/285.266
18	Codonopsine	12.879	C_14_H_21_NO_4_/267.147
19	Enigmol	13.365	C_18_H_39_NO_2_/301.297
20	7-Hexadecen-1-ol	13.430	C_16_H_32_O/240.245
21	(Z)-2-Amino-1-hydroxyoctadec-4-en-3-one	13.498	C_18_H_35_NO_2_/297.266
22	Dihydroceramide C2	13.500	C_20_H_41_NO_3_/343.308
23	6-Hydroxysphingosine	13.502	C_18_H_37_NO_3_/315.277
24	Methyl 8-[2-(2-formyl-vinyl)-3-hydroxy-5-oxo-cyclopentyl]-octanoate	13.585	C_17_H_26_O_5_/310.179
25	Dehydrophytosphingosine	13.753	C_18_H_37_NO_3_/315.277
26	14-Methyl-8-hexadecen-1-ol	13.795	C_17_H_34_O/254.261
27	Nonadecanal	13.800	C_19_H_38_O/282.291
28	Oleoyl Ethanolamide	15.294	C_20_H_39_NO_2_/325.296
29	Linoleamide	15.308	C_18_H_33_NO/279.256

**Table 5:** Docking scores.

Ligand	Free Binding Energy (Kcal/mol)	Estimated Ki
Afzelechin 3-*O*-alpha-l-rhamnopyranoside	−11.43	4.18 nM
Cucumerin A	−12.0	1.59 nM
Quercetin	−10.18	34.57 nM
Allopurinol	−5.66	70.74 uM
